# Optimization of Muscle Activity for Task-Level Goals Predicts Complex Changes in Limb Forces across Biomechanical Contexts

**DOI:** 10.1371/journal.pcbi.1002465

**Published:** 2012-04-12

**Authors:** J. Lucas McKay, Lena H. Ting

**Affiliations:** The Wallace H. Coulter Department of Biomedical Engineering, Emory University and the Georgia Institute of Technology, Atlanta, Georgia, United States of America; University College London, United Kingdom

## Abstract

Optimality principles have been proposed as a general framework for understanding motor control in animals and humans largely based on their ability to predict general features movement in idealized motor tasks. However, generalizing these concepts past proof-of-principle to understand the neuromechanical transformation from task-level control to detailed execution-level muscle activity and forces during behaviorally-relevant motor tasks has proved difficult. In an unrestrained balance task in cats, we demonstrate that achieving task-level constraints center of mass forces and moments while minimizing control effort predicts detailed patterns of muscle activity and ground reaction forces in an anatomically-realistic musculoskeletal model. Whereas optimization is typically used to resolve redundancy at a single level of the motor hierarchy, we simultaneously resolved redundancy across both muscles and limbs and directly compared predictions to experimental measures across multiple perturbation directions that elicit different intra- and interlimb coordination patterns. Further, although some candidate task-level variables and cost functions generated indistinguishable predictions in a single biomechanical context, we identified a common optimization framework that could predict up to 48 experimental conditions per animal (n = 3) across both perturbation directions and different biomechanical contexts created by altering animals' postural configuration. Predictions were further improved by imposing experimentally-derived muscle synergy constraints, suggesting additional task variables or costs that may be relevant to the neural control of balance. These results suggested that reduced-dimension neural control mechanisms such as muscle synergies can achieve similar kinetics to the optimal solution, but with increased control effort (≈2×) compared to individual muscle control. Our results are consistent with the idea that hierarchical, task-level neural control mechanisms previously associated with voluntary tasks may also be used in automatic brainstem-mediated pathways for balance.

## Author Summary

The nervous system has the ability to rapidly and flexibly coordinate many muscles and limbs to produce movements. This neuromechanical transformation must robustly achieve motor goals under the changing mechanics of the body and environment, and select one solution amongst many alternatives. What computational principles govern such decisions? Although optimality principles have predicted features of biological movement in simple models, here we show that this computational principle can robustly predict detailed experimental measures in an unrestrained, whole-body balance task. Detailed patterns of muscle activity and forces across multiple movement directions and body configurations were predicted based on interactions between musculoskeletal mechanics of the limbs, and task-level neural strategy of controlling the CoM mechanics while minimizing control effort. Moreover, similar muscle activity and forces were generated when muscles were coupled together in groups called muscle synergies, reducing the number of independent variables that are controlled. Our work is consistent with the idea that the nervous system may learn to coordinate muscles and limbs by minimizing effort in producing natural movements, and may use approximate solutions based on muscle synergies. Understanding such neural mechanisms may allow us to predict the effects of neural injury and disease on motor function.

## Introduction

Although optimality principles have been presented as a general framework for understanding motor control in animals and in humans [1], the ability of optimization to explain experimental data using high-dimensional musculoskeletal models remains largely unknown. Studies using optimization approaches have demonstrated an impressive ability to predict qualitative features of motor behaviors, such as the presence of low-dimensional muscle patterns [2], [3], and the presence of high levels of noise in some redundant degrees of freedom and low levels of noise in others [4]. Further, studies using approaches based on optimal feedback control have even predicted features such as countermovements [1], [5]. However, much of this evidence relies on biomechanical models that are abstract [1], that lack muscles [6], [7] or that have reduced degrees of freedom for computational efficiency [3], [8], [9], [10]. When complex musculoskeletal models are used to predict experimental data, the greatly increased complexity often precludes investigation of more than a single experimental condition [2], [11], which may be insufficient to discriminate different candidate control strategies or cost functions [12], [13]. Here, our goal was to test optimization as a predictive tool for understanding motor control by predicting detailed changes in experimentally-measured quantities across multiple biomechanical conditions.

**Figure 1:**
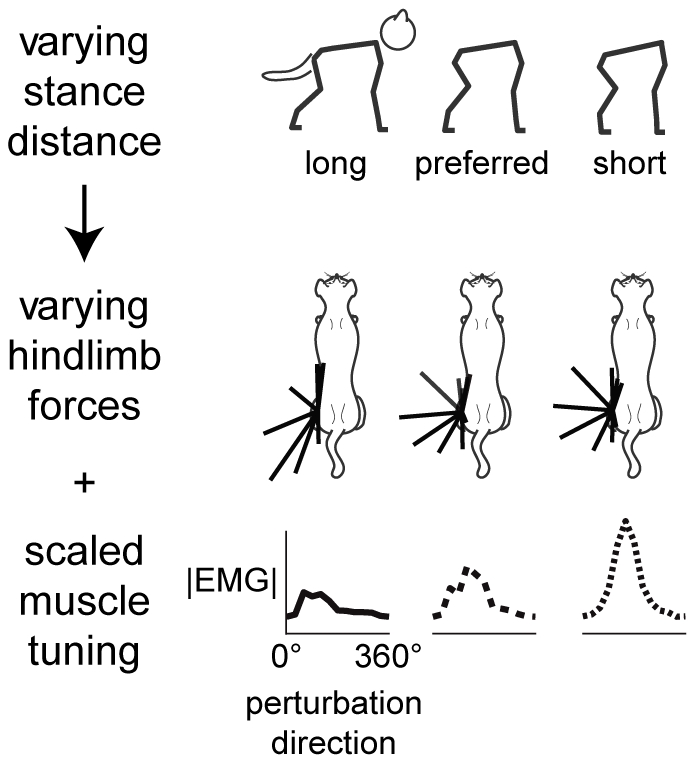
Schematic of variations in muscle activity and limb forces with altered stance distance during balance tasks in cats hypothesized to arise from neuromechanical interactions. Top to bottom: sagittal-plane kinematics, left hindlimb ground reaction forces, left hindlimb muscle tuning curves. As stance distance between the fore- and hind-limbs is decreased from left to right (top row), a wider range of ground reaction force directions is observed (middle row), as well as increased muscle activation; however, muscle tuning to perturbation direction is conserved (bottom row).

The postural response to perturbations during standing balance is a motor paradigm in which consistent patterns of motor outputs are elicited across different biomechanical contexts, but the degree to which these patterns reflect neural control or biomechanical mechanisms is unknown. To maintain balance, the center of mass (CoM), a task-level variable, must be maintained above the base of support of the feet. Robust patterns of muscle activity referred to as the automatic postural response (APR) occur about 40 ms after horizontal translations of the support surface [14] and are consistently tuned to the direction of CoM motion across different perturbation types [15], suggesting that these long-latency responses reflect task-level control of the CoM by the nervous system. This robustness is surprising given that in a quadruped, the net force acting on the CoM can be produced by many combinations of individual limb forces. Further, each limb force can be produced by many patterns of muscle activity due to muscular redundancy [16], [17]. Active ground reaction forces during the postural response (∼100 ms latency) tend to be directed along a diagonal axis either towards or away from the CoM across perturbation directions [18]. The distribution of individual limb force direction and magnitude in the horizontal plane is consistently altered by varying the distance between the fore- and hind-feet, yet surprisingly, the directional tuning of muscle activity remains intact (Figure 1) [19], suggesting that the limb force variation may be due largely to differences in biomechanical context across postural configurations.

However, we demonstrated that biomechanical constraints alone are insufficient to determine the active production of limb forces during perturbations to standing balance. Measured postural forces are nearly ten times smaller than the absolute force production capability of a detailed musculoskeletal model of the isolated cat hindlimb in all directions [20]. The diagonal orientation of the forces is not predicted from anisotropies in the force-generating capability of the limb, which is greatest in the anterior-posterior direction. Further, changes in postural force directions across biomechanical contexts cannot be attributed to alterations in the force-generating capability of the limb, as peak force directions do not change appreciably across postural configurations [21]. Therefore, here we sought to improve our predictions of experimental measures through the addition of a model of a neural control mechanism that could achieve appropriate task-level forces and moments at the CoM while coordinating redundancy across both multiple muscles and across multiple limbs.

The optimal feedback control of CoM dynamics predicts the timecourse of activity in single muscles during balance control in both quadrupeds and bipeds [22], [23], [24]; however, it remains unknown whether task-level constraints at the CoM are sufficient to predict execution-level motor patterns across multiple muscles and limbs in a complex and redundant musculoskeletal system. Such redundancy has previously been resolved by minimizing neural control effort, assumed to be equivalent to the sum squared muscle activation or sum squared motor commands [2], [13], [25], [26]. Such optimizations have been applied to predict muscle tuning curves across conditions in relatively simple or quasi-static motor tasks [2], [26], or to deduce complex muscle activation patterns from detailed kinetic and kinematic measures [27]. Moreover, effort minimization is also sometimes treated as equivalent to energy minimization, which can predict aspects of gait in simple models of locomotion in humans and other animals [28], [29], [30]. However, predicting muscle coordination in detailed musculoskeletal models by minimizing quantities like effort remains challenging [11].

Further, it has been argued that low-dimensional muscle patterns emerge from optimization of the activation of individual muscles, without explicit neural constraints on muscle activation [1], [2]. While low-dimensional patterns in the form of muscle synergy groupings have been observed experimentally [19], [31], [32], [33], [34], [35], [36], studies using planar musculoskeletal models have noted similarities in motor behaviors predicted by optimally controlling individual muscles or muscle synergies [3], [37]. Such predictions have been based on relatively simple or abstract musculoskeletal models, and thus it is not clear whether such emergent low-dimensional patterns are competent to predict forces and muscle activation patterns in more behaviorally-relevant motor tasks. It has also been argued that muscle synergies may allow for near-optimal performance with simplified computations based on a reduced number of controlled variables [37], [38], but may increase control effort due to additional coactivation [39]. However, direct comparisons of the energetic cost associated with controlling individual muscles or muscle synergies in a 3D model of a natural behavior have not been performed.

Here, we sought to identify a task-level optimization framework that could predict execution-level limb forces and muscle tuning measured in an unrestrained balance task across different biomechanical contexts. We hypothesized that features of execution-level patterns of limb forces and muscle activity reflect the minimum-effort solution for achieving appropriate forces and moments at the CoM. We compared predictions using a static quadrupedal musculoskeletal model of the cat to data from experiments. Specifically, we predicted that limb forces would be directed along the diagonal for long stance distances, and more evenly distributed in direction at short stance distance. Further, we predicted that muscle activity would be low-dimensional, and that muscle tuning to perturbation direction would scale, but not shift as postural configuration varied. By varying cost functions and task-level variables we demonstrated that the predicted outputs depended on the optimization formulation, and not simply the biomechanical constraints. Finally we compared results from optimal control of individual muscles to those based on controlling experimentally-derived muscle synergies. Our work suggests that the neural control of this natural behavior can be well described by a cost function that minimizes effort expended in the muscles in order to achieve appropriate forces and moments to stabilize the CoM. Further, our results are consistent with the idea that the computation may be implemented in a hierarchical control framework that allows for approximately-optimal motor patterns with a reduced number of controlled variables.

## Methods

### Summary

To test the hypothesis that execution-level variables reflect optimal control of task-level variables, we predicted patterns of limb forces and muscle activity in response to multidirectional postural perturbations in cats based on achieving task-level mechanics while minimizing different formulations of control effort (Table 1). Using a detailed static quadrupedal musculoskeletal model of standing balance, we first identified patterns of muscle activity that produced forces and moments at the CoM necessary to maintain balance in response to postural perturbations in twelve different perturbation directions while minimizing neural control effort (model MMe). We considered multiple postural configurations with altered stance distance between the fore- and hind-feet. We compared identified muscle activation patterns and the resulting ground reaction forces to mean values measured experimentally during the initial response. In order to demonstrate that biomechanical constraints alone could not account for the identified solutions, we demonstrated that alternate cost functions and task goals produced qualitatively different results. We compared predictions from minimum effort control of CoM force and moment to predictions from minimizing an alternative cost function designed to be a better representation of the metabolic energy used in the muscles (model MMm). Additionally, we compared predictions of controlling an alternate task-level variable, the position of the center of pressure (CoP; model MPe). Finally, to investigate whether task-level control of the CoM could be accomplished with a small number of muscle synergies, rather than with individual muscles, we constrained the muscles in the model to activate in muscle synergies adapted from previously-observed experimental data (models SMe and SMc). We estimated and compared the energetic cost, the computational cost, match to experimental data, and the dimensionality of the muscle activation patterns predicted by controlling individual muscles or postural muscle synergies.

**Table 1:** Hypothesized models of optimal task-level control.

Model	Execution-Level Variable	Task-Level Variable	Cost Function
**MMe**	muscle	CoM	muscle effort, Eq. 3
**MMm**	muscle	CoM	muscle energy, Eq. 4
**MPe**	muscle	CoP	muscle effort, Eq. 3
**SMe**	synergy	CoM	muscle effort, Eq. 3
**SMc**	synergy	CoM	synergy effort, Eq. 6

### Postural perturbation paradigm

**Figure 2:**
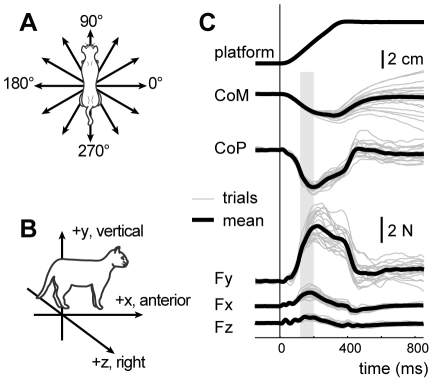
Experimental postural perturbation paradigm and example data used for model constraints and validation. *A:* Directions of translational perturbations are evenly-spaced in the horizontal plane. *B:* Coordinate system for forces and kinematics. *C:* Time traces of platform position, CoM and CoP displacement for a 60° perturbation along the direction of the perturbation, and left hindlimb ground reaction forces for 20 perturbations (cat bi) in the preferred postural configuration. The shaded region represents the initial period of active force generation due to the postural response. The CoM and CoP values in the time bin shown were used to define constraints on performance of the quadrupedal model, and individual forces across the four limbs were then compared to model predictions.

We parameterized the musculoskeletal model and assessed predicted limb forces and muscle activation patterns using previously-collected data of three cats during quiet standing and postural perturbations in multiple postural configurations [19]. The cats (bi, 2.7 kg; ru, 4.2 kg; ni, 3.5 kg) were trained to stand unrestrained with weight evenly distributed on four 8 cm-square force plates mounted on a moveable perturbation platform that could translate in any of 12 directions in the horizontal plane (Figure 2). Translations were 15 cm/s velocity and 5 cm amplitude. Data were collected in a self-selected postural configuration (preferred configuration), and in postural configurations in which the stance distance between the fore- and hind- force plates was altered. The following stance distances were examined in each of the animals: bi, 30 cm, 27 cm (preferred), 20 cm, and 13 cm; ru, 40 cm, 29 cm (preferred), 24 cm, and 18 cm; ni, 29 cm (preferred), 24 cm, and 18 cm. Stance width between the left and right force plates was 8 cm in all conditions.

We modeled muscle activity and limb forces associated with the initial period of the automatic postural response (APR) to perturbation, which can be studied as a quasi-static process. Multiple experimental and modeling studies have demonstrated that the forces during the initial portion of the APR can be attributed primarily to muscular forces [19], [40], [41]. During this period the acceleration- and velocity-dependent terms in the equations of motion are negligible so that the influence of dynamic terms on ground reaction forces is minimal [42] and the task can be approximated as quasi-static. This feature is due to the fact that there are distinct delays between the perturbation onset, the evoked muscular activity, and the subsequent active force. EMG activity due to the initial perturbation acceleration occur approximately 60 ms after the onset of the perturbation and only produces active forces at the ground after an additional 60 ms delay. Thus, there is no interaction between the perturbation acceleration and the active forces which occur during the constant-velocity, e.g. quasi-static phase of the perturbation [15]. Similarly, the acceleration of the body segments is largest while the acceleration of the platform is transmitted across all body segments [43], whereas after this period, the CoM has approximately constant horizontal-plane velocity (note the approximately constant slope of the CoM displacement during the active period indicated by the gray bar, Figure 2). Therefore, inertial forces associated with segment accelerations are not appreciable during the active response. Second, due to the relatively short latency of the active response compared to the overall motion, the posture of the animal has not changed appreciably from quiet standing at the onset of the active response. The posture of the animal affects gravitational forces, as well as torque generation via the muscle moment arm matrix. However, at the onset of the active force, the total displacement of the CoM is typically less than 1 cm and the effective tilt angle of the CoM is 1–2° [15]. Therefore the posture can be considered to be static, with no appreciable changes in gravitational forces or muscle moment arms. Therefore, our model assumes that all of the ground-reaction forces during the initial period of the APR are due to muscular activation, rather than dynamic terms.

### Quadrupedal musculoskeletal model

We created the quadrupedal musculoskeletal model by modifying and assembling four instances of an existing static, 3-D musculoskeletal model of the cat right hindlimb [20], [21]. The hindlimb model relates 31-element muscle excitation vectors 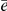 to the six-element force and moment system 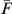 produced at the hindlimb endpoint:
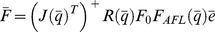
(1)where the vector 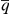 is comprised of the model's seven kinematic degrees of freedom: three at the hip, and two each at the knee and ankle, 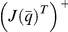 designates the Moore-Penrose pseudoinverse of the transpose of the geometric system Jacobian (*pinv.m*), 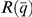 designates the moment-arm matrix, and 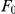 and 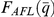 are diagonal matrices of maximum isometric forces and scaling factors based on muscle force-length properties [44]. Hindlimb model parameters are provided for each animal and experimental condition in Dataset S1. The muscles included in the hindlimb model and recorded in experimental data are summarized in Table 2. A closed-form expression for the Jacobian was identified with AutoLev software (Online Dynamics, Inc., Sunnyvale, CA, USA; currently being developed as MotionGenesis Kane) and implemented in Matlab (Mathworks, Natick, MA). The model of the left hindlimb was created by duplicating the right hindlimb model and reversing the sign of the lateral force component.

**Table 2:** Summary of muscles included in the hindlimb model and analyzed in experimental data.

Muscle name	Abbreviation	Muscle name	Abbreviation
adductor femoris	ADF	plantaris	PLAN
adductor lounges	ADL	psoas minor	PSOAS^b,n^
biceps femoris anterior	BFA^n^	peroneus tertius	PT
biceps femoris posterior	BFP^b,n,r^	pyriformis	PYR
extensor digitorum longus	EDL	quadratus femoris	QF
flexor digitorum longus	FDL^r^	rectus femoris	RF^b,n,r^
flexor hallucis longus	FHL	sartorius	SART^b,n,r^
gluteus maximus	GMAX	semimembranosus	SM^b,n,r^
gluteus medius	GMED^b,n,r^	soleus	SOL
gluteus minimus	GMIN	semitendinosus	ST^n,r^
gracilis	GRAC^b,n,r^	tibialis anterior	TA
lateral gastrocnemius	LG	tibialis posterior	TP
medial gastrocnemius	MG	vastus intermedius	VI^b^
peroneus brevis	PB	vastus lateralis	VL^b^
Pectineus	PEC	vastus medius	VM^b,n,r^
peroneus longus	PL		

Superscripts b, n, r designate muscles that were recorded in cats bi, ni, and ru, respectively.

Prior analyses demonstrated that the hindlimb model is insensitive to the pseudoinverse operation, although the choice of pseudoinverse can be particularly important in robotics applications [45], [46]. Two previous studies demonstrated that the overall hindlimb force production capability is unchanged whether one degree of freedom (hip rotation) is locked, making the Jacobian 6×6 and exactly invertible [20], or whether the pseudoinverse is used [21], because the majority of the muscles in the model have hip rotation moment arms that are small in comparison to other degrees of freedom at the hip. Further, very similar endpoint force directions are produced by the muscles in the model in these two conditions. Across muscles, animals, and experimental conditions, the average difference in predicted endpoint force direction between the hip-locked and pseudoinverse conditions was only a few degrees (2.8±5.0°, dorsal plane; 4.4±11.3°, sagittal plane). These results are consistent with recent experimental results in which similar mappings between muscle forces and endpoint forces and torques were identified when mechanical degrees of freedom were locked or freed [46].

Because a detailed musculoskeletal model of the forelimb was unavailable, we approximated the forelimb by modifying the hindlimb model into a vertical strut that transformed muscle activation to vertical force. Although the forelimbs do not always contribute to horizontal-plane forces during the postural response [18], they contribute non-negligible vertical forces, of magnitudes several times larger than their horizontal force magnitudes. There also may be less potential for horizontal-plane forces to be produced by the extensor muscles in the cat forelimb because the morphology is more columnar than that of the hindlimb. Therefore, we approximated the forelimb as a transformation from muscle activity to vertical force by eliminating all rows of Equation 1 except for the row corresponding to vertical force.

The transformation from muscle activation to CoM force and moment in the quadrupedal musculoskeletal model was found using the forces from each limb and the approximate location of the CoM. Resultant CoM force was calculated as the sum of the individual limb forces. Resultant CoM moment was calculated as the sum of the vector cross products between the vectors from the CoM to the limb endpoints and the limb forces. Limb endpoint moments were assumed to make negligible contributions to the net moment at the CoM. The net force and moment at the CoM due to individual limb forces 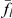 is thus:
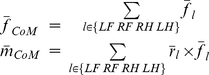
(2)Where 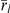 designates the vector from the CoM to the endpoint of limb 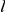
_._ The transformation from muscle activation to force and moment at the CoM was formulated as a 6×124 matrix equation for each postural configuration and animal relating muscle activation levels (31 muscles in each limb, for 124 total) to the 6D CoM force and moment.

### Musculoskeletal model parameterization

**Figure 3:**
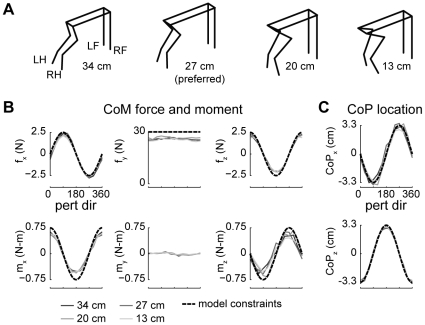
Kinematic and kinetic constraints used in the optimal control models. *A:* Kinematics of the musculoskeletal model parameterized to cat bi at four stance distances. LH, left hindlimb; LF, left forelimb; RF, right forelimb; RH, right hindlimb. *B:* average forces and moments at the CoM in each perturbation direction. Solid lines indicate experimental data, dashed lines indicate task-level constraints used in models MMe, MMm, SMe, SMc. *C:* average displacement of the CoP in each perturbation direction. Solid lines indicate experimental data, dashed lines indicate task-level constraints used in model MPe.

We identified joint angles in the musculoskeletal model that best approximated the recorded kinematics of each cat during quiet standing in each postural configuration (Figure 3A). Positions of kinematic markers located on the platform and the left sides of the body were collected at 100 Hz during each trial for each cat. Locations of joint centers were estimated from marker positions by subtracting off joint radii, skin widths, and marker widths. The joint angles that minimized the squared error between the sagittal- and frontal-plane angles of the femur, shank, and foot in the model and in the background-period kinematics of each trial of each cat were identified using numerical optimization (*fmincon.m*) [20]. All residual segment angle errors were ≤10^−4^°. Joint angles were averaged across like trials. Muscle moment arm values and fiber lengths were determined with SIMM software (Musculographics, Inc., Santa Rosa, CA) and averaged across like trials.

We approximated the location of the CoM with respect to the feet in the musculoskeletal model separately for each cat in each postural configuration based on kinematic data and morphological parameters. For all conditions, the CoM was assumed to be located midway between the limb endpoints in both the anterior-posterior and medial-lateral directions. The height of the CoM above the plane of the feet was estimated from kinematic data and morphological parameters separately for each cat in each postural configuration. Across postural configurations, average CoM heights for each cat were (mean ± SD): bi, 12.6±0.4 cm; ru, 15.2±0.4 cm; ni, 12.7±0.8 cm.

### Minimum-effort control of CoM force and moment (model MMe)

To determine whether minimum-effort task-level control of the CoM could predict execution-level limb forces and muscle activity, we first identified patterns of muscle activity in the musculoskeletal model that produced forces and moments at the CoM similar to observed values while minimizing squared muscle activation (model MMe). Task-level constraints on CoM force and moment were based on average values from experimental data (Figure 3B). Average limb forces and CoM positions during the active period 120–200 ms after perturbation onset [19] were combined to estimate the average forces and moments at the CoM for each perturbation direction and postural configuration of each animal. Moments generated at the limb endpoints were assumed to make negligible contributions to the net CoM moment. Because values were similar across animals and postural configurations, a single set of average CoM forces and moments that was considered representative for all animals was then created and used as the optimization constraint: net horizontal-plane forces directed in the perturbation direction of 2.5 N magnitude, net vertical forces of 30 N, and net pitch-roll moments of 0.75 N-m magnitude directed perpendicular to the perturbation direction. CoM yaw moment was left unconstrained. Muscle activation patterns that satisfied task-level constraints could not be identified analytically without violating physiological bounds on muscle activation [44]. Therefore, optimizations were formulated as quadratic programming problems (*quadprog.m*) to identify muscle activation patterns that satisfied task-level constraints while minimizing total squared muscle activation:
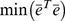
(3)Where 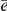 designates a vector containing the activity levels of all muscles in the model (124). Additional constraints ensured that the activation levels of each muscle were in the interval (0,1) and that vertical ground reaction forces were ≥0. Separate optimizations were performed for each animal, postural configuration, and perturbation direction.

### Minimum-energy control of CoM force and moment (model MMm)

To investigate whether similar force predictions could arise from optimization criteria other than the minimum effort criterion used in model MMe, we next altered the cost function to better approximate metabolic energy consumption in the muscles, in terms of Joules/second, than minimizing Equation 3, but without the added complexity of Hill-type muscle models [47]. In single muscle fibers, metabolic energy usage (Joules/sec) is proportional to stress [48], equivalent to muscle activation in the model used here [44]. We assumed that the number of fibers in a muscle, and therefore its energy consumption, is proportional to its mass. Therefore, we performed additional optimizations with constraints and methods identical to the first model formulation, but minimizing total squared muscle activation weighted by muscle mass:
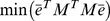
(4)Where 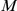 is a diagonal matrix of muscle masses. Masses for each muscle are included in Dataset S1. The majority of muscle masses (23/31 hindlimb muscles) were taken from the literature [49]. Because muscles for which no data were available were typically small, these masses were all set to a common low-midrange value.

### Minimum-effort control of CoP location (model MPe)

To investigate whether the minimum-effort control of an alternate task-variable could predict similar limb forces, we tested a formulation similar to model MMe, except constrained to match displacements of the CoP in each perturbation direction, leaving the net force at the CoM unconstrained. Some studies of sagittal-plane balance in humans have suggested that the location of the CoP is the task-level variable controlled during balance [50]. Task-level constraints on CoP displacement were based on average values from experimental data (Figure 3C). The average displacement of the CoP at the midpoint of the active period in each perturbation direction for each postural configuration of each animal was calculated from the four vertical forces [15]. Similar to the first model formulation, a single set of corrections in CoP location (3.3 cm in magnitude and directed opposite the direction of the perturbation) was created and used as task-level constraints in the optimizations.

### Minimum-effort control of CoM force and moment using postural muscle synergies (models SMe and SMc)

Next, to determine whether task-level control of the CoM could be accomplished with a small number of muscle synergies, rather than individual muscles, we constrained the muscles in each limb of the musculoskeletal model to activate in 5 muscle synergies based on muscle synergy force vectors previously observed in the same animals during the balance task [19]. The model [21], assumes that the activation of each muscle results from the additive combination of a few muscle synergies 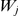, recruited by scaling coefficients 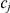. The activation level of the muscles in the model is therefore:
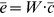
(5)where each element 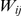 of 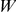 represents the activation of the 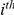 muscle by the 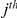 muscle synergy, restricted to be within the interval (0,1), and the elements of scaling coefficients 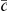 are restricted to be greater than zero. Five muscle synergies and related ground reaction force vectors were previously extracted from experimental data of each animal using nonnegative matrix factorization [19]. The muscle synergy patterns used in the model were subsequently derived by identifying patterns of muscle activation in the hindlimb model that could produce each ground reaction force vector while minimizing squared muscle activation (Equation 3) [21]. Identical muscle synergies were used in each limb and in all postural configurations. The constraints and solution method in this formulation were very similar to model MMe, with the exception that muscle synergy activation levels 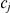 were identified rather than muscle activation levels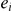. Synergy activation levels were constrained to be positive with respect to a level that created a background net vertical force. We considered two different cost functions in optimizations of muscle synergy control. Optimizations were performed that minimized muscle effort, (model SMe), as in Equation 3, but with the addition of muscle synergy constraints:
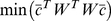
(6)Further, to determine whether optimal solutions could be identified entirely in reduced-dimension space, optimizations were also performed (model SMc) that satisfied task-level constraints on CoM force and moment while minimizing sum squared muscle synergy activation:
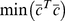
(7)


### Assessment of predicted limb forces

We calculated goodness-of-fit between predicted left hindlimb and right forelimb forces and experimental data from each animal across experimental conditions. Because vertical force (VF) magnitudes are several times larger than horizontal force (HF) magnitudes, they were analyzed separately. We compared predicted left hindlimb (LH) HF direction, LH HF magnitude, LH VF magnitude, and right forelimb (RF) VF magnitude with experimental data. R^2^ values for each force component were calculated across perturbation directions for each postural configuration for each animal and subjected to two-way ANOVAs (postural configuration×animal) evaluated with a significance level of α = 0.05 adjusted with a Bonferroni correction for multiple comparisons (α = 0.0125) to determine whether the predictive ability of each formulation depended on the experimental condition. Left hindlimb HF magnitudes in perturbation directions that loaded the hindlimb (0° through 90°) were also subjected to two-way ANOVA (postural configuration×animal) evaluated at α = 0.05 to determine whether magnitudes decreased as postural configuration was varied. R^2^ values predicted by the different model formulations were subjected to three-way ANOVAs (postural configuration×animal×formulation) and evaluated at the Bonferroni-corrected level of α = 0.0125.

### Assessment of predicted muscle activity

We compared predicted muscle tuning curves to mean values from each animal and experimental condition. Mean values of EMG were calculated during the initial burst of muscle activity 60–140 ms after perturbation onset, and averaged across like trials. We compared the scaling and shifting in predicted tuning curve peak values across postural configurations to changes observed in data. Muscle tuning curves were normalized to maximum values observed in the preferred postural configuration of each cat. The peak magnitude and perturbation direction of each muscle tuning curve in each postural configuration of each animal was identified and expressed as a change from the preferred configuration value, either as a magnitude change, or as a direction change in degrees. In tuning curves with more than one peak, we tracked the peak value that was dominant in the preferred postural configuration. Tuning curve scaling was assessed by regressing peak values onto postural configuration (L,P,S,SS) and comparing the resulting regression coefficients for each cat and model. Tuning curve shifting was assessed by calculating the maximum change in peak direction across postural configurations. These values were then subjected to one-way ANOVA evaluated at α = 0.05 to determine whether shifts predicted by each model were comparable to observed values.

We assessed the dimensionality of muscle activation patterns predicted by models MMe, SMe, and SMc using a simple criterion based on principal components analysis (PCA). As we were primarily interested in comparing muscle activity pattern dimension predicted by controlling individual muscles (MMe) versus that predicted by controlling postural muscle synergies (SMe, SMc), we used a simple criterion that excludes components that contribute less variance than any individual variable in the original dataset [51], [52]. Vectors of predicted left hindlimb muscle activation were assembled into matrices arranged with perturbation directions along the rows and muscles along the columns. Separate matrices were assembled for each postural configuration and animal. The dimensionality of each matrix was then estimated as the number of eigenvalues of the data correlation matrix ≥1.0. Dimensionality estimates were pooled across animals and postural configurations and subjected to one-way ANOVA at a significance level of α = 0.05 to determine whether the formulations predicted similar muscle activity dimensionality. Dimensionality estimates from each model were compared to 5, the previously reported value [19]. Comparisons were performed with t-tests at a significance level of α = 0.05, adjusted with a Bonferroni correction for multiple comparisons to α = 0.0167.

### Comparison of effort and computation time required for controlling muscles and postural muscle synergies

We compared the total control effort required for controlling individual muscles (MMe) versus that required for controlling postural muscle synergies (SMe, SMc). The control effort required for the muscle activity predicted by each model formulation was calculated with Equation 3. Values were normalized to 100% of the value predicted by optimal muscle control in the preferred postural configuration of each cat. We then performed one-way ANOVA on the resulting values, at a significance level of α = 0.05, to determine whether the three formulations predicted similar sum-squared muscle activity. We estimated the computational cost predicted by the three formulations by measuring and comparing the time required for each formulation to identify muscle activity patterns in all perturbation directions in each experimental condition. Resulting values were subjected to one-way ANOVA at a significance level of α = 0.05, to determine whether the three formulations required similar computation time.

## Results

### Summary

Task-level constraints on CoM force and moment or CoP location were satisfied by all of the models considered, but each predicted different patterns of muscle activity and limb forces, demonstrating the high level of redundancy of the quadrupedal musculoskeletal system. Experimentally-measured horizontal plane limb forces at preferred stance distance were predicted by task-level control of CoM forces and moments using either the minimum-effort or the minimum-energy cost functions (models MMe and MMm), whereas solutions predicted by control of CoP control (MPe) differed substantially. However, differences between forces and moments predicted by models MMe and MMm were revealed when limb forces were examined across postural configurations; although MMe solutions varied in magnitude across stance distances in a similar fashion to experimental measures, MMm solutions did not predict any qualitative differences in limb forces across stance distances. Limb forces similar to MMe predictions were found when a muscle synergy constraint was enforced (models SMe and SMc). In all three models that matched experimental limb forces across postural configurations (MMe, SMe, SMc), muscle tuning directions were found to be invariant across postural configurations, similar to experimental data, resulting in low-dimensional overall muscle activity patterns. However, using muscle synergies derived from experimental data (SMe, SMc) allowed better predictions of activity in flexors, some of which were not activated in the independent muscle coordination conditions (MMe). Finally, control effort increased by several times, but the time required for the quadratic programming search was decreased, when muscle synergies were controlled (SMe, SMc) rather than individual muscles (MMe).

### Models MMe and MMm predicted individual limb forces in the preferred postural configuration

**Figure 4:**
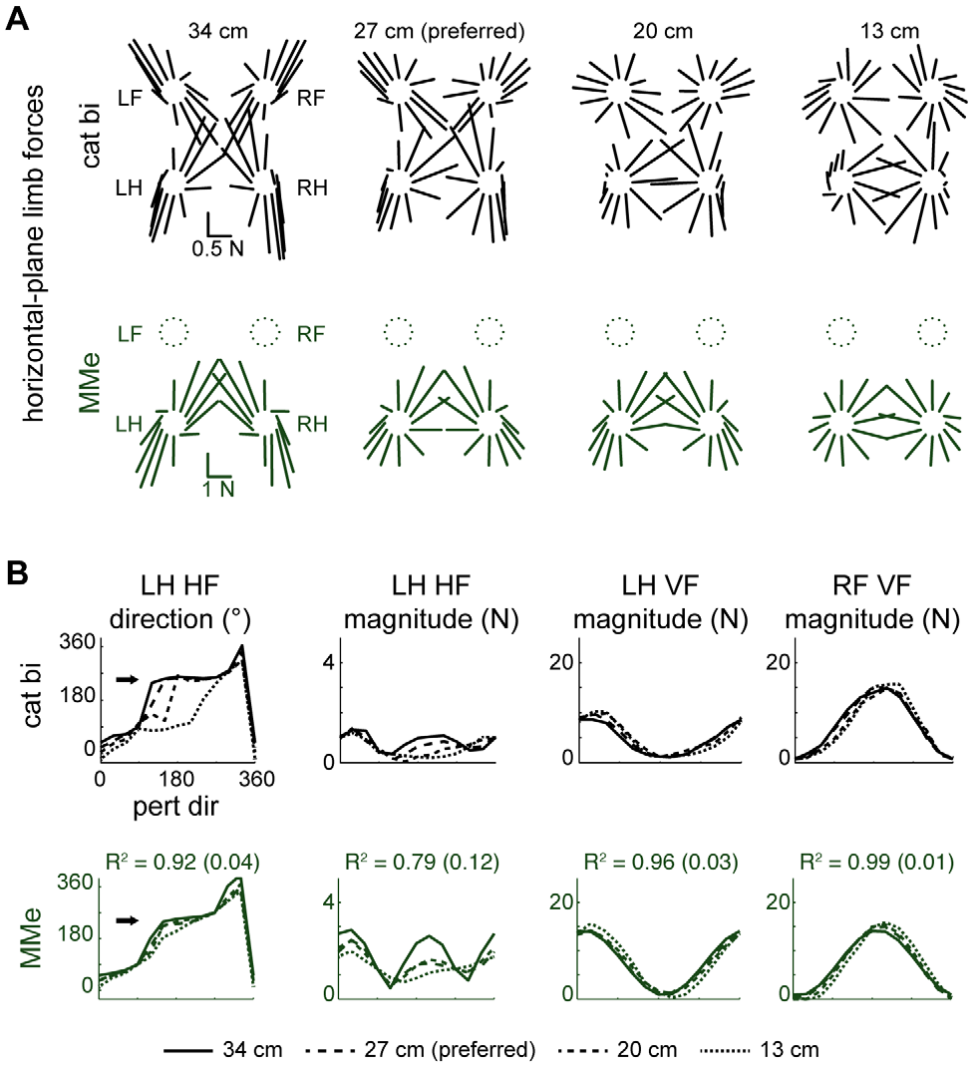
Limb forces predicted by optimal task-level control of CoM force and moment. *A:* average horizontal plane forces observed in each postural configuration of cat bi (black) compared with model MMe predictions (green). Force vectors are drawn for each limb (clockwise from top left: LF, left forelimb; RF, right forelimb; RH, right hindlimb; LH, left hindlimb) with their origins offset in the direction of platform motion. Stance distance decreases from left to right. Predicted forces were directed towards and away from the CoM, characteristic of the force constraint strategy described previously [18] at longer stance distances (34 and 27 cm), whereas a wider range of force directions was observed at shorter stance distances (13 cm) [53]. *B:* comparison of average and predicted limb force components in polar coordinates. HF, horizontal force; VF, vertical force. Predicted horizontal plane forces reproduced the region of invariant force directions for perturbation directions that unloaded the hindlimb (180° to 270°) observed at longer stance distances (34 and 27 cm) (arrows).

**Figure 5:**
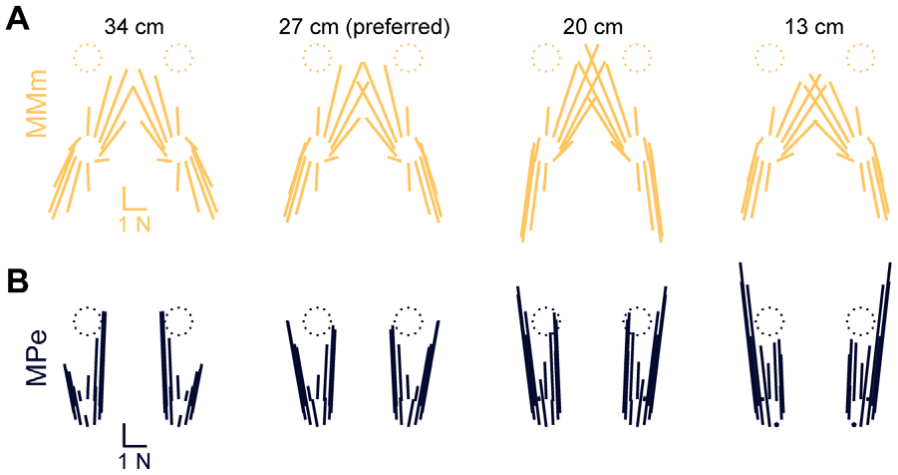
Predicted horizontal plane forces obtained by altering the cost function and the task level variable. *A:* model MMm predictions (yellow). Note that unlike MMe predictions (Figure 4A), MMm predictions are approximately constant as stance distance decreases. Compare changes between 34 cm and 13 cm to changes in Figure 4A. *B:* model MPe predictions (blue). Note that predicted forces are near the anterior-posterior axis, the strongest axis of force production in the isolated hindlimb [20] in all perturbation directions and postural configurations.

Although we did not explicitly try to match experimentally-measured limb forces with the model, task-level control of CoM force and moment using either the minimum effort (model MMe) or minimum energy (MMm) cost functions nonetheless predicted horizontal plane forces directed towards and away from the CoM characteristic of the force constraint strategy described previously [18] in the preferred postural configuration (Figure 4A,B, 27 cm; Figure 5A, 27 cm). Across all perturbation directions, predicted left hindlimb HF directions were similar to data (MMe: mean R^2^ = 0.89±0.08, P<1e-3; MMm: 0.84±0.08, P<1e-3). In perturbation directions that loaded the left hindlimb (0° to 90°), predicted HF forces were directed towards the CoM, similar to data (data: mean direction 56±28°; MMe: 67±19°; MMm: 76±8°). In perturbation directions that unloaded the left hindlimb (180° to 270°), horizontal-plane forces were directed away from the CoM, again similar to data (data: mean direction 263±9°; MMe: 254±13°; MMm: 258±6°).

Left hindlimb HF magnitudes predicted by both cost functions varied as bimodal functions of perturbation direction similar to experimental data, particularly in loaded perturbation directions (MMe: mean R^2^ = 0.94±0.09; MMm: 0.91±0.05). Fits of left hindlimb HF magnitudes across all perturbation directions were reduced somewhat because of the small recorded force magnitudes in the unloaded perturbation directions (MMe: mean R^2^ = 0.77±0.29; MMm: 0.48±0.09). Maximal left hindlimb HF magnitudes were observed near 30° perturbations that loaded the hindlimb and minimal values for perturbations towards 120°, near the opposite diagonal axis. Average hindlimb HF magnitudes in perturbation directions where the left hindlimb was loaded (0° to 90°) were 1.2±0.4 N in data vs. 2.4±0.9 N and 3.3±0.3 N, in the MMe and MMm models, respectively. Absolute predicted HF magnitudes were larger than recorded values, which was necessary in order to account for the absent contributions of the forelimbs.

VF magnitudes predicted by both cost functions exhibited a realistic exchange between the forelimbs and hindlimbs as a function of the perturbation direction (R^2^>0.98). For perturbations diagonally to the right (near 30°), left hindlimb vertical forces were maximal (data: 11.4±3.4 N; MMe: 12.4±1.9 N; MMm: 12.3±1.8 N), whereas recorded right forelimb vertical forces were near minimal (data: 3.8±2.5 N; MMe: 2.1±2.3 N; MMm: 2.0±2.4 N). Both cost functions predicted complete unloading (0 N) of the left hindlimb and right forelimb in some cases, whereas the minimum vertical reaction forces observed in data were 1.0 N in the hindlimb and 0.6 N in the forelimb.

### Model MMe, but not model MMm, predicted variations in limb forces across postural configurations

Differences between the predictions of models MMe and MMm became apparent when other postural configurations were considered. Variations in left hindlimb HF direction and magnitude were observed across stance distances similar to data [53] in model MMe, but not in model MMm. As stance distance was decreased, a wider range of HF directions was observed in MMe but not MMm (e.g., compare changes between 27 cm and 13 cm in Figure 4A versus Figure 5A). Similarly, HF magnitude from longest to shortest stance had a greater decreasing trend in MMe (−9±22%, P<0.25) than MMm (−4±8%; P<0.55) in unloaded directions (180° to 270°). However, neither reached the degree of HF magnitude change observed experimentally (−59±31%; P≪0.001) in unloaded directions. Over all directions, HF magnitude fits to data were significantly higher (P<0.001) in MMe compared to MMm (Table 3). Moreover, HF magnitude fits were similar across postural configurations in MMe, but were significantly decreased at shorter stance distances in MMm (*P*<0.0002).

**Table 3:** Summary of average limb force R^2^ values predicted by each model formulation.

	Left Hindlimb						Right Forelimb	
	HF Dir		HF Mag		VF Mag		VF Mag	
**MMe**	0.86	(0.06)	0.60	(0.32)	0.97	(0.02)	0.98	(0.01)
**MMm**	0.81	(0.08)	0.30*	(0.26)	0.96	(0.02)	0.98	(0.02)
**MPe**	0.36	(0.15)	0.23	(0.20)	0.93	(0.03)	0.95	(0.03)
**SMe**	0.90	(0.08)	0.50	(0.27)	0.96	(0.04)	0.91	(0.13)
**SMc**	0.91	(0.06)	0.58	(0.31)	0.91	(0.05)	0.87	(0.10)

R^2^ values are presented as mean (SD) across animals and postural configurations. HF Dir, horizontal force direction; HF Mag, horizontal force magnitude; VF Mag, vertical force magnitude.

*: significant variation across postural configurations (*P*<0.0125).

Differences in forces across postural configurations were due to the fact that MMe favored recruitment of large muscles whereas MMm favored recruitment of small muscles. Large muscles that produce downward and backward endpoint forces relative to the limb axis were preferentially activated in MMe. When stance distance is shortened, the force rotates to have a more vertical orientation, thus reducing the component of force in the horizontal plane [19], [21]. In contrast, smaller muscles produce forces with relatively small elevations in the horizontal plane, so that horizontal plane force components are relatively constant as stance distance is shortened. Compared to MMe, model MMm reduced the activation of large antigravity muscles by several times (LG, mass 12.4 g, 1/3×; VL, 19.6 g, 1/4×) and increased the activation of small muscles by 5–1000 times (PSOAS, 4.0 g, 4×; SOL, 4.03 g, 20×; VI, 4.39 g, 5×; PT, 1.06 g, 1000×).

### Model MPe predicted unrealistic limb forces in all postural configurations

Unlike experimental data, model MPe predicted HF directions near the strongest axis of force production in the isolated hindlimb [20] in all perturbation directions and postural configurations (Figure 5B) to achieve task-level constraints on CoP location. Because CoP location is measured about the projection of the CoM on the ground, predicted CoM forces and moments deviated significantly from experimental measures (peak deviations: anterior force, 18.7±2.0 N; rightwards force, 1.9±0.4 N, roll-right moment, 0.2±0.1 N-m; pitch-up moment, 2.5±0.3 N-m). Although VF magnitudes predicted by model MPe were similar to data (R^2^>0.86), HF direction fits were poor (R^2^ = 0.36±0.15), and CoM-directed horizontal-plane forces were never observed. Instead, average left hindlimb HF directions were 90±3° and 98±3° for perturbation directions that loaded, and unloaded the left hindlimb respectively, near the direction of maximum force production of the hindlimb [20]. CoP control requires only modulation of VF magnitude across all four legs; large horizontal forces result from the fact that the minimum-effort muscle activation pattern to produce a vertical force component also has a very large horizontal component. These predictions were similar when the minimum energy cost function (Equation 3) was used (not shown).

### Models SMe and SMc predicted active unloading limb forces superior to model MMe

**Figure 6:**
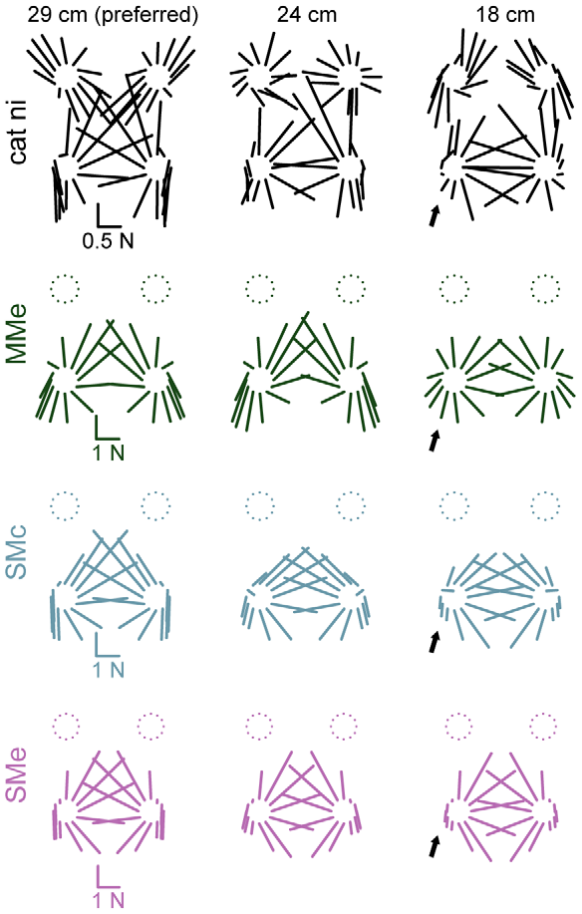
Predicted horizontal plane forces obtained by controlling experimentally-derived muscle synergies versus individual muscles. Top to bottom: average horizontal-plane forces observed in each postural configuration of cat ni (black), predictions of models controlling individual muscles: MMe (green), or muscle synergies: SMc (blue), and SMe (purple). Arrows highlight significant force magnitude reductions observed in data, SMc, and SMe, but not in MMe.

**Figure 7:**
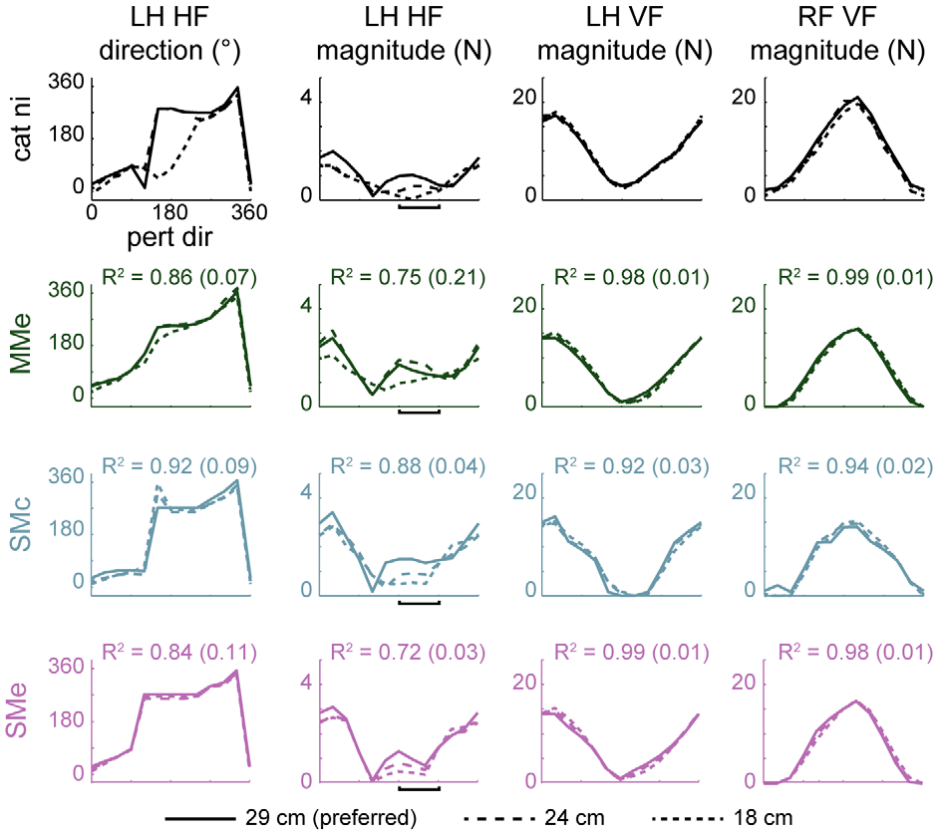
Comparison of limb forces predicted by controlling experimentally-derived muscle synergies rather than individual muscles in polar coordinates. Data correspond to horizontal-plane forces shown in Figure 6. Colors as in Figure 6. Note that LH HF magnitudes in perturbation directions that unloaded the left hindlimb (horizontal bars, 180° to 270°) exhibited a monotonic decrease in models SMc and SMe from the preferred (29 cm, solid lines) to the shortest (18 cm, shortest dashed lines) stance distance similar to data that was not predicted in MMe.

Adding muscle synergy constraints (models SMe and SMc) resulted in limb forces that were similar overall to predictions of model MMe (Table 1); however, SMc additionally predicted a reduction in HF magnitude at shorter postural configurations that was comparable to the data (see arrows in Figure 6). As in MMe predictions, muscle synergy control models predicted characteristic HF directions towards (SMe, 83±100°; SMc, 68±80°) and away (SMe, 254±45°; SMc, 255±42°) from the CoM; however, visual inspection suggested that HF directions were more dispersed compared to MMe. Superior to MMe predictions, both muscle synergy control models predicted statistically-significant decreases in HF magnitudes in unloaded perturbation directions as stance distance decreased from preferred to shortest (SMc, −31±39%, P≪0.0001; SMe, −4±44%, P<0.04), although decreases were still less than those observed experimentally (−59±31%). VF magnitudes were predicted well in both the left hindlimb and right forelimb in SMe and SMc (R^2^ = 0.93±0.05), although MMe predictions remained superior (P<0.001). As in MMe predictions, both the left hindlimb and right forelimb completely unloaded in some cases for SMe and SMc (Figure 7). In some perturbation directions of the shortest postural configuration of cat bi (SMe, 5/132 total; SMc, 6/132) VF magnitude constraints were relaxed to allow CoM constraints to be achieved; these were excluded from further analysis.

### Models MMe, SMe, and SMc predicted cosine muscle tuning comparable to experimental data

**Figure 8:**
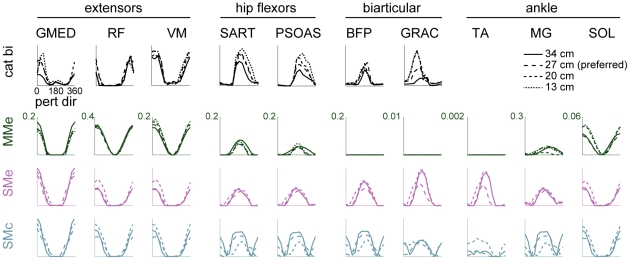
Examples of left hindlimb muscle tuning to perturbation direction observed in data and predicted by optimal task-level control using individual muscles or experimentally-derived muscle synergies. Top to bottom: experimental data, predictions of models MMm, SMe, and SMc. Colors as in Figure 6. All models predicted smooth cosine muscle tuning to perturbation direction similar to experimental data, particularly in morphologically simple extensors (e.g., VM, SOL) and in some flexors (e.g., PSOAS). However, some flexors were recruited only when muscle synergies were controlled rather than individual muscles. Compare BFP, GRAC, TA in MMe vs. SMe. Some multifunctional muscles were more difficult to predict; e.g., unlike experimental results [15], MG was recruited with pattern similar to a flexor muscle in all models, with ankle extension being provided by extensor-tuned SOL. Biarticular muscle SART is listed as a hip flexor because it is implemented as such in the musculoskeletal model.

All models that predicted realistic limb forces across postural configurations (MMe, SMe, SMc) predicted smooth cosine muscle tuning to perturbation direction similar to experimental data, particularly in morphologically simple extensors (Figure 8). Experimentally-observed tuning curves from left hindlimb extensors were typically cosine-shaped and centered around rightwards perturbations (0°) with approximate widths of 90°–120° at half-maximum (e.g., VM, GMED). Models MMe, SMe, and SMc made similar predictions for several extensors, including GMAX, GMED, VI, VM, VL, and SOL. Recruitment was not identical across models; for example, hip extensor BFA was recruited with similar tuning in SMe and SMc, but only in 1/3 cats in MMe. Some multifunctional extensors were more difficult to predict; for example, hip flexor/knee extensor RF was recruited in posterior/rightwards perturbations towards 330° experimentally, but predicted tuning curves (MMe, SMe, SMc) were centered about 0°. Ankle extensor/knee flexor MG was recruited with tuning curves centered near 180° by all models, unlike experimental results [15]; this tuning was similar to that observed in flexors, suggesting that the function at the knee might be dominating, with ankle extension being provided by extensor-tuned SOL. Ankle extensor/knee flexor LG was also recruited with tuning near 180° (1/3 cats, MMe) or with bimodal tuning to leftwards and rightwards perturbations (3/3 cats, SMe, SMc).

In some cases, the activation of flexor muscles was predicted by models SMe and SMc, but not by model MMe. Although some flexors were recruited with realistic cosine tuning about 180° in MMe, including PSOAS and SART (Figure 8), others were recruited in SMe and SMc but were never recruited in MMe. Ankle flexor TA was recruited with realistic cosine tuning to leftwards perturbations only in SMe, and only in cat bi. Some bifunctional muscles with flexor contributions were recruited in SMe and SMc but not in MMe. For example, hip extensor/knee flexor BFP was never recruited in MMe, but was recruited in 3/3 cats in SMe and SMc. Hip extensor/knee flexor GRAC was similar (2/3 cats, SMe, SMc; 0/3 cats, MMe), although predicted tuning curves were phase shifted somewhat from the anterior/leftwards tuning observed experimentally. Although hip extensor/knee flexor STEN was never recruited in MMe, it was recruited in SMe and SMc, but with either a bimodal (2/3) or extensor pattern (1/3).

**Figure 9:**
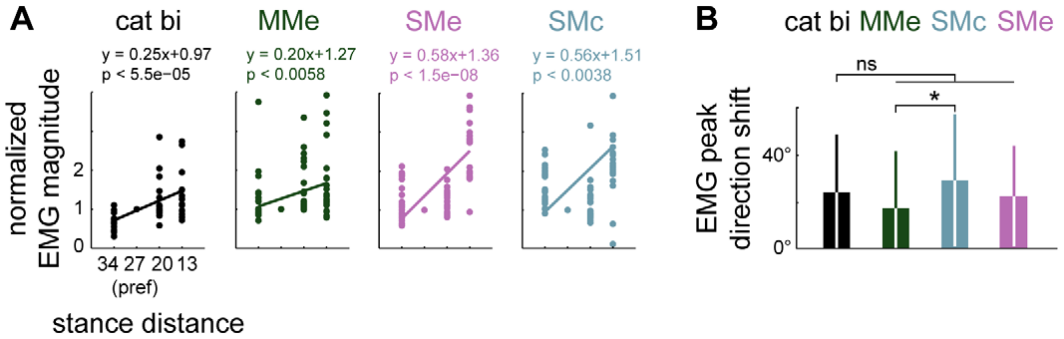
Observed and predicted changes in muscle tuning curve magnitude and direction across postural configurations. *A:* Comparison of muscle tuning curve magnitude scaling across postural configurations observed in cat bi with scaling predicted by models MMm, SMe, and SMc. Data points for individual muscles are shown as filled circles. *B:* Comparison of muscle tuning curve peak direction shift across postural configurations observed in cat bi with direction shifts predicted in models MMm, SMe, and SMc. Note that although SMc predicted increased tuning curve shifting compared to MMe, none of the models predicted significantly increased shifting compared to experimental data. ns, p>0.05; *, P<0.05; ANOVA, post hoc tests.

Models MMe, SMe, and SMc all predicted muscle tuning curves that scaled in magnitude and shifted as stance distance was decreased comparable to experimental data (Figure 9). EMG peak magnitude increased as stance distance was shortened both in experimental data (regression slopes of 0.25, P<0.0001, bi; 0.10, ni; 0.10, ru) and in model predictions (MMe, 0.19±0.01; SMe, 0.26±0.27; SMc, 0.22±0.29; all P<0.022). Tuning curves predicted by all three models exhibited shifting with postural configuration that was not significantly different (P>0.05) from recorded values (average variation in peak tuning direction in data, 24±24°; MMe, 18±24°; SMe, 23±21°; SMc, 29±28°), although model SMc predicted increased tuning curve shifting compared to predictions of model MMe.

Models MMe, SMe, and SMc all predicted low dimensional muscle activity patterns, with muscle synergy control predicting lower dimensional EMG than individual muscle control. Patterns of left hindlimb muscle activity predicted in MMe were characterized by 4.3±0.5 principal components across cats and postural configurations, significantly higher (P<0.0001) muscle synergy control predictions (SMe: 3.2±0.6; SMc: 3.1±0.7). Dimensionality estimates from models MMe, SMe, and SMc were all significantly lower (P<0.0001) than 5, the number of muscle synergies previously identified in the balance task [19].

**Figure 10:**
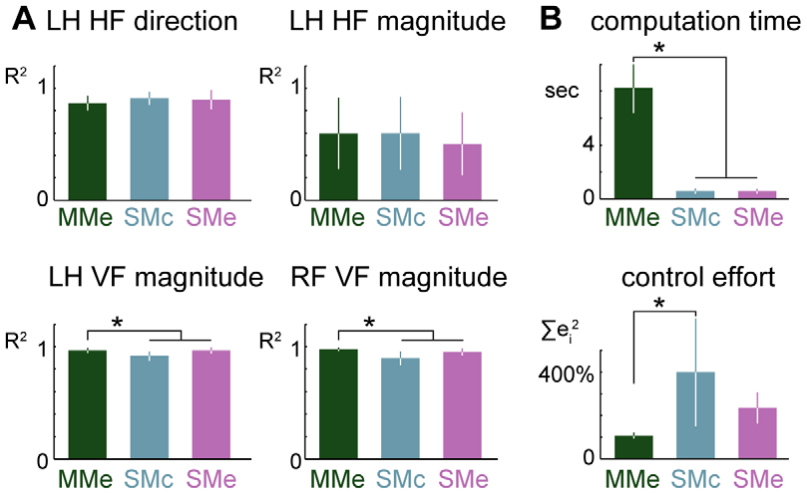
Comparison of predicted fits to limb force data, computation time and control effort required for task-level optimal control formulations. Controlling experimentally-derived muscle synergies predicts similar force outputs, but with reduced computation time and increased control effort compared to controlling individual muscles. A: comparison of fits to limb force data predicted by models MMe, SMe, and SMc. LH, left hindlimb; RF, right forelimb. Colors as in Figure 6. B: comparison of average computation time and average sum-squared left hindlimb muscle activation predicted by models MMe, SMe, and SMc. Muscle activation values for each cat are normalized to 100% of the amount predicted by model MMe in the preferred postural configuration.

Models of muscle synergy control required more control effort, but less computation time during the quadratic programming search, than model MMe (Figure 10B). Using muscle synergy control reduced the computation time by a factor of 8 compared to MMe (P≪0.001) whereas control effort increased 2–4 times (P<0.0005). Post hoc analyses revealed a significant contrast between the control effort required for the MMe and SMc models (P<0.05).

### Constraining endpoint moments to zero did not affect forces predicted by model MMe

To test whether model MMe might be predicting unrealistic endpoint moments, MMe optimizations in the preferred postural configuration of each animal were repeated with additional constraints such that the moments at each limb endpoint were limited to zero. This formulation predicted fits to experimentally-observed left hindlimb HF directions that were similar to those of the MMe model (P<0.83, paired *t*-test). Due to the additional constraints, 5/12 optimizations of cat *Ni* failed to converge and were excluded. Convergence failures occurred in the same conditions in ten repetitions of these optimizations.

## Discussion

Our results demonstrate how optimality principles can be used to understand how the nervous system may distribute effort across redundant muscles and limbs to achieve task-level goals during the automatic postural response, a natural motor behavior. Importantly, this work demonstrates that optimality principles can predict experimental data in the context of a detailed musculoskeletal model. We demonstrate that achieving task-level constraints on the forces and moments at the CoM while minimizing the control effort to the muscles can simultaneously resolve redundancy at the level of both muscles and limb forces during the initial portion of the automatic postural response. Moreover, by examining a rich repertoire of experimental conditions, we were able to distinguish amongst candidate task-level variables and effort cost functions, which often generated indistinguishable predictions in a single biomechanical context. Predictions were further improved by imposing constraints based on experimentally-derived muscle synergies and muscle synergy force vectors, demonstrating the feasibility of muscle synergies as physiological mechanisms for the implementation of near-optimal motor solutions, as well as suggesting additional costs and constraints that were not included in our original optimization framework. These results are consistent with the idea that the hierarchical, task-level neural control mechanisms previously identified in cortically-mediated tasks may also be relevant in understanding brainstem-mediated motor tasks.

### Optimization predicts detailed motor patterns across biomechanical contexts

Although prior studies demonstrated that temporal patterns of activation of individual muscles during balance could be predicted from task-level optimal control of CoM dynamics and control effort, they did not address the partitioning of control effort across redundant muscles or limbs. Temporal patterns of individual muscle activity during balance can be predicted from an optimal tradeoff between minimizing CoM excursion and control effort in both humans and cats [23], [24]. However, previous models of CoM control during balance have eliminated redundancy by examining single-plane movements, as well as by controlling the joints with torques [7], [54], [55], [56], [57], [58], [59] or single muscles [23], [24]. In contrast, we focused on predicting spatial patterns of activity at the initial timepoint of the CoM feedback response in order to understand the coordination of multiple muscles and limbs across multiple perturbation directions spanning the horizontal plane.

Here, we found that detailed patterns of muscle activity and limb forces across biomechanical contexts were predicted from interactions between a common optimization framework – achieving task-level constraints while minimizing effort – and the changing properties of the musculoskeletal system. Prior studies demonstrated that the properties of single-limb biomechanics [20], [21] were insufficient to predict the force directions observed across multiple postural configurations [14], [18], [53], leaving the role of biomechanics in determining this behavior unclear. These results suggest that control effort costs influence the way that the nervous system distributes effort across the redundant musculature when different combinations of muscles can realize the constraints of the task, and that the characteristic changes in forces observed during the balance task emerge as optimal patterns of distribution are applied in different biomechanical configurations. Moreover, constraints on net CoM mechanics allowed both muscle and limb force redundancy to be simultaneously resolved by minimizing control effort [2], [13], [26], eliminating the need to explicitly minimize limb force [25], [60].

Our results also demonstrate the feasibility of muscle synergies to produce approximately optimal motor patterns in the context of a detailed model in a realistic motor task. Multiple studies have demonstrated that muscle synergies might be a feasible and effective way for the nervous system to produce movement [61], [62], [63], and that the control of muscle synergies can closely approximate the optimal control of individual muscles, particularly in planar or idealized tasks [37], [64], [65]. We found that muscle synergy control was sufficient to achieve the task constraints, in some cases recreating the activation of flexors that was not well-predicted by minimizing the activation of individual muscles. However, in general, solutions from optimal muscle control and muscle synergy control were broadly similar, consistent with the results of other studies [2], [66]. For example, extensor muscle activity and the limb forces in perturbations for which the hindlimb was loaded were well-predicted whether the activity of individual muscles or of muscle synergies was optimized. Although our study does not resolve the debate over whether low-dimensional muscle activation patterns reflect optimal patterns of individual muscle control or explicit muscle synergy constraints, these results demonstrate the feasibility of muscle synergies for the implementation of near-optimal motor solutions in a realistic motor task.

Taken together, the results of this and previous studies are consistent with the idea that the temporal and spatial patterning of muscle activity during the automatic postural response can be well-described by a hierarchical optimal control framework. Hierarchical optimal control is based on the idea that higher levels of the nervous system operate on increasingly abstract variables, such as CoM kinematics, while relying on lower-level controllers to locally control high-dimensional musculoskeletal dynamics [67], [68]. We hypothesize that the high-level representation is critical because multiple studies have demonstrated that lower-level kinematic variables such as joint angles are insufficient to predict the activation of individual muscles during balance control, whereas CoM kinematics robustly predicts which muscles will be activated [15], [54], [69], [70], [71], [72]. Such a hierarchical structure may be required in neural control structures due to neural conduction and computation delays. One idea proposed for the low-level control architectures is that they might implement local feedback control to linearize the nonlinear, fast dynamics of the musculoskeletal system, or implement other regulatory functions [68], [73]. Our concept of a muscle synergy is proposed as a transformation between high-level task goals and low-level dynamics, that may be parameterized to optimally actuate musculoskeletal mechanics [64] or to provide stability [74], but not necessarily to function as a controller *per se*. We speculate that CoM feedback may be used to recruit muscle synergies, and in support of this, a recent study in human balance control demonstrated that CoM kinematics are sufficient to describe the temporal recruitment of postural muscle synergies throughout complex perturbations [75]. Despite the various differences, the similarity between solutions arising from optimization of the activity of individual muscles and optimization of the activity of muscle synergies are consistent with the idea that muscle synergies may reflect mid- or low-level control structures within a general hierarchical optimal control scheme for movement.

### Model interpretation, validation, and limitations

While control of the CoP was sufficient to explain the results of previous studies that considered a limited range of biomechanical conditions, we were able to compare CoP and CoM as task-level variables by examining their ability to predict individual limb forces across multiple directions of perturbation. Both the CoM and CoP have been proposed as controlled variables for balance control [50], [70], but control of CoP involves fewer constraints and is based on the control of vertical and not horizontal limb forces. These candidate control variables have typically been investigated in models of only a single plane of movement [7], [23], [24], [54], [55], [56], [57], [58], [59], where they may make indistinguishable predictions. Here, forces predicted by the two candidate task-variables were similar for the direction of primary limb loading in which lateral forces were small. Predictions of sagittal-plane limb forces were also similar across both models (MMe vs. MPe) in the directions across all directions in which the limb was loaded. Given the anisotropic force generation characteristics of the hindlimb [20], it seemed plausible that the control of vertical forces could be sufficient to determine shear forces as well. However, the models produced qualitatively different horizontal plane forces, suggesting that additional constraints on CoM moment and force were necessary to predict the observed force patterns in a quadruped. It is possible that CoM and CoP control are indistinguishable in sagittal plane balance control in humans where force generation is primarily in the vertical direction [76], [77]. However, the predictions of CoP control are likely to break down when significant horizontal place forces are required such as in our quadrupedal model, or in medial-lateral human balance control. Further, CoP control in human and robot walking has been limited to quasi-static conditions [57], [78], [79], whereas more dynamic conditions suggest that angular momentum about the CoM due to CoM moments is an important control variable [80], [81], [82], [83], [84], [85]. Importantly, these results demonstrate that the observed muscle activity patterns and forces could result from an optimization framework in which task-level goals are specified, independent of individual limb forces.

We noted that different cost functions produced qualitatively different patterns of limb forces, demonstrating that the experimentally measured patterns are not simply due to musculoskeletal constraints, but indeed depend upon the nature of the optimization framework. Prior studies have found that multiple cost functions could produce similar results [13], [86], suggesting that solutions may be qualitatively determined by biomechanical constraints, independent of any optimization framework or control policy. In contrast, our study and other recent studies demonstrate that some cost functions can be eliminated based on their robustness across a wider range of experimental conditions [12], [25]. Here, minimization of muscle effort (MMe) versus energy (MMm) predicted similar horizontal plane forces in the preferred postural configuration, but not in short or long stance configurations. In order to more precisely determine a physiological cost function inverse optimization approaches could be used [25], [87], [88]. However, it is unlikely that composite cost functions based on weightings between MMe and MMm [25], [89] would improve fits to recorded muscle activity (e.g. absent flexors, SOL recruited rather than MG), as both cost functions strongly penalize muscle coactivation. Neither are these differences likely to be resolved using alternative cost functions such as minimization of signal dependent noise, which predicts muscle activity patterns similar to minimization of control effort [90].

To further investigate either the task-level variable or the cost function would require implementation of task-level control within a dynamic musculoskeletal model. Although balance control is a dynamic task, we were able to use a static musculoskeletal model to examine the force-sharing problem at a specific instant in time during the postural response that is most amenable to description by a quasi-static model (see Methods). Here we sought only to reproduce the net CoM forces and moments observed in the initial postural response, which in turn can be predicted by an optimal feedback control model in a low-dimensional biomechanical model [22], [23], [24]. Integrating an optimal controller with a realistic musculoskeletal model would allow us to test various optimal control models for dynamic balance control, which might implicate criteria relevant to the balance task beyond the control cost formulations presented here. Specifically, considering the longer time constants required to deactivate versus activate muscle [91] would likely improve model predictions by encouraging activation of the flexors. Similarly, rewarding recruitment of muscles with fast fiber types would likely encourage the ankle extensor function of MG (primarily fast muscle fibers), over that of SOL (primarily slow muscle fibers; [92]). Other criteria such as those related to mechanical stability might also be used to explain the absent coactivation [16]. For example, arm impedance is increased in unstable environments, likely requiring additional coactivation [93]. It is possible that these costs could be incorporated within an optimal control formulation penalizing response time in a tradeoff with costs such as control effort, as optimal control models without fixed terminal time have recently been developed for motor tasks [94], [95], [96]. A dynamic model would also allow for further refinement of the task variable. Although we were able to differentiate between CoM and CoP, the current model cannot differentiate between CoM and some other candidate task-level variables – for example, translations of the CoM along the anterior-posterior axis – since a static model ignores inertial contributions such that an equivalent moment can be computed about any point.

We consider it unlikely that adding additional detail to either the hindlimb or the forelimb models would appreciably influence the forces predicted here. Based on the high level of similarity in the force production capability between the static hindlimb model used here and previous dynamic models, it is unlikely that including a linearized dynamic model with the mass matrix would appreciably influence the results. Previous linearized and fully dynamic versions of the hindlimb model that include the mass matrix have demonstrated nearly identical force production capability to the static model used here [20], with force production capability biased along the anterior-posterior axis [74], [97]. Based on earlier versions of the present model and experimental results, it is also unlikely that including a detailed forelimb model would appreciably influence the predicted forces. A previous model that included forelimbs as hindlimbs with reflected anterior-posterior force production capability did not fundamentally change the forces predicted by model MMe [98] in the preferred and long postures. However, as the stance distance shortens, the geometry of the forelimbs in a real animal becomes increasingly like that of a vertical strut, whereas the hindlimbs remain flexed, breaking the symmetry of the forces between the fore- and hind-limbs Although the fore-hind force asymmetry in the shorter postures was not very pronounced in these particular animals modeled here, in some cases the forelimb forces are not elongated at all [14], [18], suggesting that the forelimbs can be very well approximated as vertical struts in these conditions.

### Neural implications for muscle synergies and hierarchical control

Significant electrophysiological evidence exists for the neuroanatomical substrates required for the hierarchical, task-level neural control mechanisms investigated by this and other studies. While we and others have demonstrated that muscle activity and movements can be described by mathematical tools like optimization, these techniques do not explain how such relationships and computations are achieved within the nervous system [99]. Importantly, electrophysiological evidence from both cortically-mediated as well as brainstem-mediated motor tasks exists to support the idea that the hierarchical, task-level control frameworks suggested here may describe aspects of the organization of the neural substrates for motor control. For example, electrophysiological evidence demonstrates that task-level variables such as the direction of the limb endpoint are represented in motor cortex during reaching [100], [101]. Although lesion studies demonstrate that the balance task considered here does not require the cortices [102], [103], similar task-level representations are found in brainstem, where neurons in the pontomedullary reticular formation respond equivalently to perturbations of different limbs [104], [105]. Electrophysiological evidence also demonstrates that increasingly abstract representations of the motor periphery are assembled in increasingly higher levels of the nervous system. For example, higher-level representations of limb length and orientation, rather than individual joint angles, are encoded in the dorsal root ganglia and dorsal spinocerebellar tract [106], [107]. Muscle synergies may describe how task-level representations are mapped to execution-level activity of motoneurons, via the divergent projections to multiple muscles that have been identified at various levels of the nervous system [108], [109], [110], [111]. For example, both cortical and brainstem neurons project to multiple motoneurons, or to spinal interneurons [112] whose activity has been shown to reflect the patterns of muscle synergies rather than individual muscles [113].

These results support the hypothesis that muscle synergies may be important physiological mechanisms for the implementation of near-optimal motor solutions with a reduced number of controlled variables. The original concept of the muscle synergy hypothesis was that it would offer computational “simplification” due to the large numbers of independent variables that must be simultaneously controlled by the nervous system [114]. In our study, using muscle synergies significantly decreased the search time the optimization algorithm required to identify a motor solution, similar to a previous report [64]. This search time decrease illustrates the possible benefits of a reduced dimension solution space during gradient-based searches, although the computational mechanisms in the nervous system are certainly different than a computer. Stochastic search approaches, for example, might realize less benefit from reducing the dimension of the solution space. Moreover, the results do not imply that the nervous system is re-optimizing the cost function *de novo* every time the motor task is presented [25], but instead are consistent with the idea that optimal motor solutions could be refined over the course of motor learning and adaptation. Such refined solutions could be encoded within the nervous system in sparse representations that use small number of neurons at any given time. Sparse representations have been hypothesized to increased storage capacity in associative memories and increased energy efficiency [115] as well as accelerate motor learning. For example, a neural-network model demonstrated accelerated motor learning with decreases in the number of independent neural commands [38]. However, this interpretation may be somewhat controversial, as other evidence demonstrates that sparse motor representations based on muscle synergies may slow the learning of motor tasks for which the library of available muscle synergies is inappropriate [116]. We speculate that muscle synergies implement a transformation from task-level goals to muscle activation patterns that is computationally similar to a lookup table that is assembled over motor learning, the structure of which likely reflects the statistics of the behavioral repertoire as well as the motor system [117]. Similar to the arguments advanced for sparse coding of sensory inputs, we speculate that muscle synergies are reinforced over the course of motor learning through biologically-plausible local learning rules (e.g. “cells that fire together wire together”). Through such learning rules, simple model neurons can learn the principal components of their synaptic input weightings [118]. We speculate that groups of muscles would be reinforced, rather than individual muscles, because the function of individual muscles (in this case, the output force) may vary depending on the activity of the other muscles in the limb [119].

We speculate that the increased control effort required when using experimentally-derived muscle synergies versus individual muscles may be physiologically reasonable, particularly if considerations beyond energy efficiency are important in balance control. Whereas prior work demonstrated that similar efficiency could be found by controlling individual muscles or muscle synergies developed from optimality criteria [64], [65], we show that controlling experimentally-derived muscle synergies requires additional control effort. Although minimizing energetic cost may be critical in some contexts, particularly in ongoing movement tasks like locomotion over evolutionary timescales [28], [29], [30], we speculate that in discrete tasks like the balance responses presented here strictly effort-minimal solutions may not be necessary. For example, in discrete arm posture tasks, subjects can be cued to maintain high levels of coactivation out of habit even at levels of muscle activation that are considerable proportions of maximal voluntary contraction [120]. The forces observed during balance are well within the boundaries of the absolute musculoskeletal capabilities [21], and the magnitudes of the individual muscle activations predicted by model MMe were moderate, as proportions of MVC (notice that the scale maxima in Figure 8 vary between 0.002 and 0.4). Thus the additional effort cost predicted by muscle synergy control may be physiologically plausible. The fact that experimentally measured co-activation is absent in the MMe model predictions further suggests that the physiological state does not necessarily correspond to the minimum effort solution. We speculate that muscle synergies may be organized to implicitly account for criteria related to the dynamic response described above (e.g. fiber type, etc.). Particularly in balance control, using more than the absolute minimum amount of muscle activation required to achieve stability may be advantageous.
